# Mucus Hypersecretion in Chronic Obstructive Pulmonary Disease and Its Treatment

**DOI:** 10.1155/2023/8840594

**Published:** 2023-07-06

**Authors:** Binay Kumar Shah, Bivek Singh, Yukun Wang, Shuanshuan Xie, Changhui Wang

**Affiliations:** ^1^Department of Respiratory Medicine, Shanghai Tenth People's Hospital, Tongji University School of Medicine, Shanghai 200072, China; ^2^Tongji University School of Medicine, Shanghai 200092, China

## Abstract

Most patients diagnosed with chronic obstructive pulmonary disease (COPD) present with hallmark features of airway mucus hypersecretion, including cough and expectoration. Airway mucus function as a native immune system of the lung that severs to trap particulate matter and pathogens and allows them to clear from the lung via cough and ciliary transport. Chronic mucus hypersecretion (CMH) is the main factor contributing to the increased risk of morbidity and mortality in specific subsets of COPD patients. It is, therefore, primarily important to develop medications that suppress mucus hypersecretions in these patients. Although there have been some advances in COPD treatment, more work remains to be done to better understand the mechanism underlying airway mucus hypersecretion and seek more effective treatments. This review article discusses the structure and significance of mucus in the lungs focusing on gel-forming mucins and the impacts of CMH in the lungs. Furthermore, we summarize the article with pharmacological and nonpharmacological treatments as well as novel and interventional procedures to control CMH in COPD patients.

## 1. Introduction

Chronic obstructive pulmonary disease (COPD) is a pathological entity with heterogeneous and multisystem involvement characterized by a persistent airflow limitation due to enhanced chronic inflammatory response to noxious particles or gases, resulting in progressive loss of lung function and increased morbidity and mortality [1]. COPD greatly reduces patients' quality of life (QOL) and is the third leading cause of death worldwide [2]. Underdiagnoses remain a common problem due to less awareness of lung function testing in some clinicians, and therefore its prevalence is projected to increase in the coming decades [3]. The death rate of COPD increased by 11.6% in the past 30 years, from 2.8 million in 1990 to more than 3.23 million in 2019 [2, 4]. About 90% of COPD deaths under 70 years of age occurred in low- and middle-income countries [2]. Based on a population survey in China, China has a higher prevalence (8.2%–13.7%) vs. a global age-standardized prevalence of 3.2% (male)/2.0% (female) with a higher mortality rate [5].

COPD includes emphysema and chronic bronchitis (CB) with features of chronic mucus hypersecretion (CMH). CMH is defined by a medical history of chronic cough and sputum production with an increase in goblet cells, enlarged submucosal glands, and mucus production, leading to airway obstruction [6]. Patients with CMH are more likely to have increased severe airway bacterial colonization, frequent and severe exacerbation, reduced lung function, and deterioration of their health status as compared with patients without CMH. It is unknown whether clinical features due to CMH affect the response to COPD treatment, so understanding the impact of CMH may help to predict treatment response and potentially promote the development of new therapies [7].

## 2. Airway Mucus Expression

### 2.1. Anatomy and Physiology of Mucus Hypersecretion

The intrapulmonary airway consists of two types of cells: ciliated and secretory cells. Based on their microscopic appearance, secretory cells are further divided into Clara cells, goblet cells, and serous cells [8]. Secretory cells release mucus, antimicrobial, immunomodulatory, and protective molecules [9]. The mucus consists of a mixture of transudative fluid and secretions from the surface epithelium and submucosal glands. It is predominantly composed of water (95%) and macromolecular glycoproteins known as mucins (2%–3%) with smaller components of proteoglycans (0.1%–0.5%), lipids (0.3%–0.5%), proteins, and DNA [10]. Mucus in the airways is made up of a polymeric matrix of large, oligomeric, gel-forming glycoproteins, called mucin, with a protein backbone composed of a variable number of tandem repeats rich in proline, threonine, and serine as well as cysteine-rich regions at the amino and carboxy terminals resembling bottle-brush appearance [11]. Mucins have a molecular weight between 2 × 10^6^ and 40 × 10^6^ Da and are composed of 50%–85% carbohydrates [12, 13]. In addition to its physiological role in humidifying the airway, mucus also acts as a physical barrier against infections and toxins, facilitates mucociliary transport, and contributes to the innate defense system of mucosal immunity.

Mucus has largely been implicated in health and disease. It possesses unique biophysical features, including viscoelasticity and a self-healing capability [14]. Constant cilia beating propels the viscoelastic gel layer from the lower airway to the larynx. Owing to this mechanism, pathogens and inhaled particles arriving at the upper airway can be expelled by coughing or swallowing [15]. This host defense mechanism of the lung is known as mucociliary clearance (MCC). In determining the ability of an agent to promote clearance of secretions, both MCC and cough clearance need to be addressed [16].

### 2.2. Mucin Types

**Figure 1:**
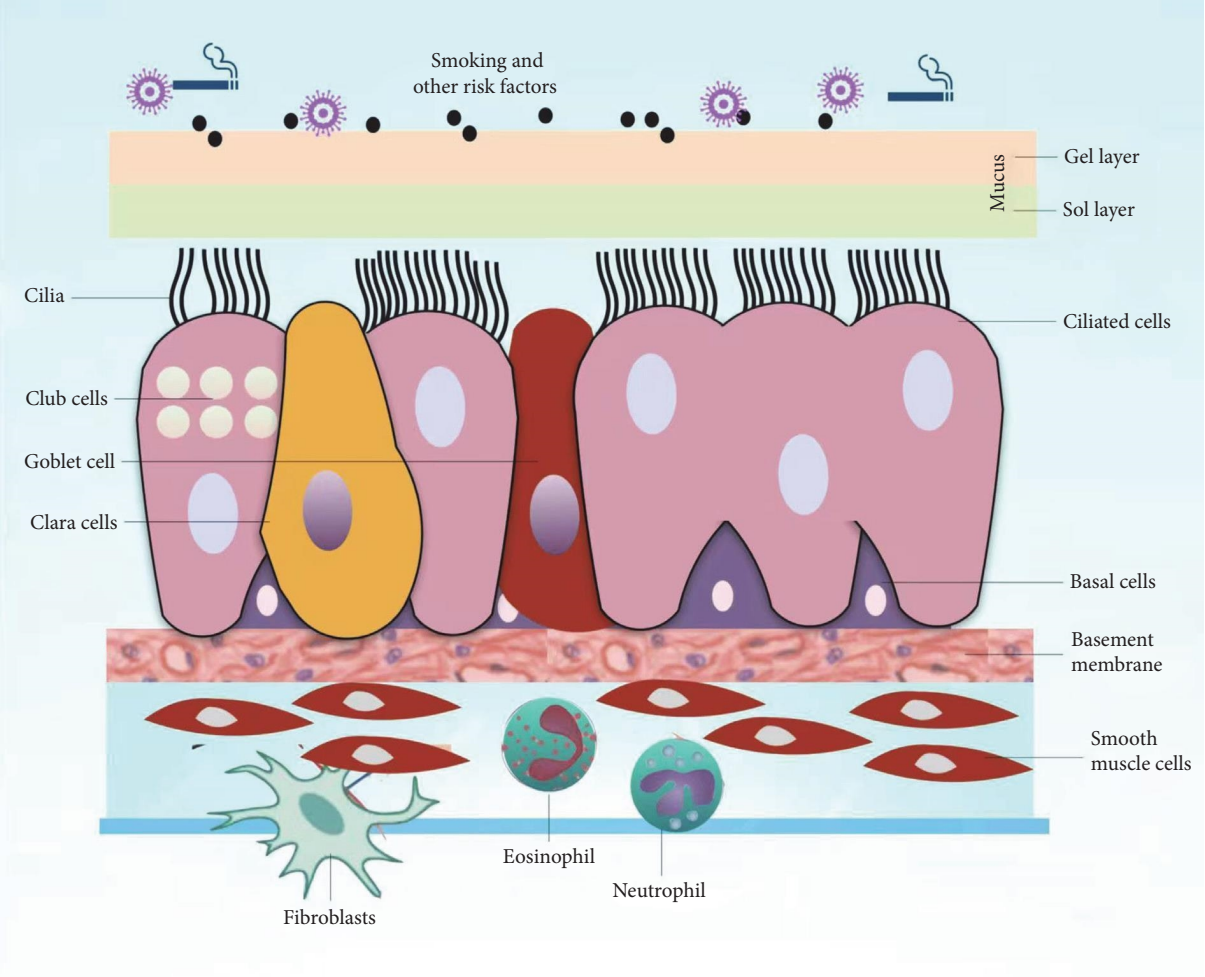
Model of airway mucus epithelium: goblet cells and submucosal glands secret mucin, which forms mucus on the surface of the airway. It contains an upper gel layer and lower sol layer, between which is a surfactant layer present which helps in lubrication and transfer of energy from beating cilia to propel out inhaled pathogens and particles by the process known as mucociliary clearance.

The airway luminal surface is coated with a multiphase mucus film composed of an upper gel layer and a lower liquid sol layer [17]. The upper gel layer, also known as the mucus layer, consists of water, mucins, lactoferrin, and various peptides, and the lower sol or periciliary liquid layer mainly consists of water (Figure 1). The two layers act like a defense barrier known as the “cilia-mucus blanket” [18]. There is a surfactant layer between the mucus gel and periciliary sol layer, which acts as a lubricant and also facilitates the transfer of energy from beating cilia to mucus [19].

Twenty-one different human mucins genes are categorized based on the backbone of their proteins, which are represented by a MUC gene (MUC1, MUC2, MUC3A, MUC3B, MUC4, MUC5AC, MUC5B, MUC6, MUC7, MUC8, OVGP1, MUC12, MUC13, EMCN, MUC15, MUC16, MUC17, MUC19, MUC20, MUC21, and MUC22), of which 14 are expressed in the respiratory system (Table 1). Different types of mucins with their chromosome locations are presented in Table 1 [20, 21]. The mucin family is divided into two subfamilies: secreted mucin and tethered cell surface-associated mucin. Secreted mucin consists of two nonpolymeric glycoproteins (MUC7 and MUC8) and five oligomeric gel-forming mucins (MUC2, MUC5AC, MUC5B, MUC6, and MUC19). The remaining three mucins are oviductal glycoprotein 1 (formerly known as MUC9), endomucin (also known as MUC14), and MUC22, in contrast to the 11 tethered cell surface-associated mucins (MUC1, MUC3A, MUC3B, MUC4, MUC12, MUC13, MUC15, MUC16, MUC17, MUC20, and MUC21) [22, 23].

MUC5AC and MUC5B are gel-forming mucins, which form the most abundantly secreted mucins in the respiratory tract, accounting for about 75% of all mucins, mainly responsible for the rheological properties of mucus, making up the glycoprotein component [24]. MUC5AC is mostly produced in the proximal airway of healthy individuals by surface goblet cells, while MUC5B is produced by submucosal glands and surface secretory cells throughout the airway [25]. These mucins are released in both normal and under stress conditions. Individuals with chronic respiratory disorders have higher MUC5AC and MUC5B levels in their airways and sputum. MUC5AC is primarily metabolized by the goblet airway epithelium, and an abnormal amount or quality of MUC5AC may impair airway function, leading to serious airway diseases, including COPD [26].

### 2.3. COPD Pathogenesis Associated with Mucus Hypersecretion

In normal situations, the pseudostratified ciliated bronchial epithelium contains a mixture of goblet, ciliated, and basal cells. Exposure to cigarette smoke or pathogens may induce goblet cell hyperplasia, decrease number of ciliated cells, and ultimately increase mucus production. Reduced airflow caused by the mucus-clogged airway contributes to the pathogenesis of COPD, seriously affecting the workability and QOL of the patients. Nearly 50% of COPD patients have CMH, with 3.5 fold higher risk of death than those without CMH [27].

### 2.4. Smoking and COPD

Smoking is a complex mixture of free radicals and other oxidants, which may cause an imbalance in oxidants/antioxidants in COPD [28]. Cigarette smoke can cause direct damage to the lung matrix components such as collagen and elastin. Certain chemicals, including uric acid, glutathione, vitamin E, and ascorbate, are decreased in smokers, which is found to be associated with the severity of COPD exacerbation [29]. Smoking results in mucus dysfunction, which has severe negative effects on the structure and function of the cilia by activating ErbB receptors and impairing the cystic fibrosis (CF) transmembrane conductance regulator function. In addition, the pro-inflammatory activity of cigarette smoking increases mucin synthesis and decreases mucus hydration and clearance [30]. Cigarette smoking comprises a variety of carcinogens, including particulate matter, reactive chemicals, and other organic compounds, among which acrolein can strongly stimulate mucin production [31].

### 2.5. Infection and COPD

Studies have shown that increased mucin formation and decreased luminal fluids in COPD reduce airway mucus clearance, thus increasing the risk of airway infection, inflammation, and fibrosis. The sputum from 25% to 50% of COPD patients carries *Haemophilus influenzae*, *Pseudomonas aeruginosa*, *Streptococcus pneumoniae*, *Moraxella catarrhalis*, and other bacteria or bacilli, which can trigger the body to produce more mucus [32]. In addition to Gram-negative bacteria, Gram-positive bacteria such as staphylococcus aureus and streptococcus pyogenes can trigger MUC2 gene transcription in epithelial cells [33]. Increased mucus production during COPD exacerbation is often linked to *H. influenzae*. A nontypeable *H. influenzae* upregulates the MUC5AC gene via TLR2-MyD88-dependent p38 MAPK (mitogen-activated protein kinase) pathway [34]. The infection rate increases with the disease severity, and COPD exacerbation is associated with the acquisition of new bacterial strains.

### 2.6. Alveolar Cells and COPD

The pulmonary alveoli are coated with two distinct types of cells, namely type Ⅰ and type Ⅱ pneumocytes. The former type accounts for 95% of the surface area of lung alveoli, while the latter type covers only 5% of the area and is comparatively resistant to damage [35]. In situations where typeⅠcells undergo damage, typeⅡcells undergo proliferation, migration, and dispersion along the depleted basement membrane surface. This process leads to the reformation of the epithelium, followed by differentiation into typeⅠcells. Thus it may help to restore the alveolar epithelial barrier. TypeⅡcells are responsible for the synthesis, storage, and secretion of pulmonary surfactant, which serves to decrease surface tension within the alveoli and promotes the stabilization of alveolar units, thereby facilitating optimal gas exchange. In addition, they produce diverse cytokines and proteins that have the ability to alter the inflammatory and oxidative stress response, as well as hinder the proliferation of fibroblasts and the synthesis of collagen, both of which are involved in the development of COPD [36].

### 2.7. Body Weight and COPD

Individuals diagnosed with COPD are more susceptible to the development of obesity due to reduced levels of physical activity and prolonged use of glucocorticoids. Prior studies have indicated approximately 65% of individuals with COPD exhibit excess weight or obesity [37]. While the correlation between obesity and a rise in mortality and morbidity in chronic disease has been well documented, studies have identified a potential protective association between obesity and COPD patients. The term “obesity paradox” describe the inverse correlation between body mass index (BMI) and chronic illness. It has been observed that a higher BMI can have significant protection on the outcome of COPD, while a lower BMI level may accelerate the decline of lung function. Also, overweight (BMI 25–29.99)/obese (BMI > 30) patients exhibit a notable increase in systemic inflammation, particularly in tumor necrosis factor-alpha (TNF-*α*), interleukin-6 (IL-6), IL-8, and leptin plasma levels, as well as 3.3 times higher C-reactive protein as compared to normal-weight patients. They exhibit a higher average length of hospital stay and utilization of invasive and noninvasive ventilation, indicating a greater strain on the healthcare system [38].

### 2.8. Immunometabolism of Cells Involved in COPD

The complex interaction between the metabolism and immune response is a crucial factor in the pathogenesis of COPD. Patients in different stages of COPD exhibit a gradual elevation of systemic leptin, a hormone with cytokine-like properties that is primarily secreted by adipocytes. This hormone has been demonstrated to play a significant role in the development of COPD by enhancing Th1/Th17 proinflammatory responses and at high concentrations hindering T cells' ability to engage in glycolysis and to generate regulatory T cells. The deprivation of these immunoregulatory pathways in COPD instigated the hyperstimulation of effector T cells, which in turn escalated inflammation, ultimately resulting in a gradual deterioration of lung function. Leptin plays a significant role in the enhancement of both innate and adaptive immunity. It directly stimulates the phagocytic activity of macrophages and potentiates the production of different cytokines that are typically involved in the pathogenesis of COPD [39].

### 2.9. *Neutrophil Elastase* (*NE*) *and Epidermal Growth Factor Receptor* (*EGFR*) *Singling Pathway*

The correlation between the number of neutrophils in sputum and decreased pulmonary function suggests a close relationship between neutrophilic inflammation and airway mucus obstruction [40]. Patients with chronic airway illnesses have a large amount of NE, a serine protease produced by neutrophils, which can ultimately result in mucus obstruction [41], damage the cilia, and impair their function, leading to mucin production, secretion, and release, secretory cell metaplasia and hyperplasia within the airway. MUC5AC mRNA levels are raised by NE, which improves mRNA stability. Recent investigations have also discovered that MUC5AC gene expression is induced by NE via reactive oxygen species (ROS) [42]. Additionally, NE has been linked to mucin synthesis via EGFR activation and TNF-*α* secretion, which induces EGFR expression in airway epithelial cells [43]. Mucus hypersecretion in COPD is affected by inflammatory neutrophils, NE, ROS, EGFR, and neutrophil necrosis [44].

### 2.10. Cytokines Induce Mucin Production

Cytokines, particularly T helper 2 cytokines such as IL-4, IL-9, IL-13, IL-17A, etc., have been demonstrated as potential stimulators of mucus production [45, 46]. A study postulated that the sterile alpha motif-pointed domain-containing Ets transcription factor (SPDEF) may participate in controlling prostate growth and cancer [47]. However, another study has revealed that SPDEF is also expressed in the airway, where it controls the differentiation of airway epithelial cells. IL3, STAT6, SPDEF, and MUC5AC are the primary singling pathways causing the differentiation of epithelial cells to goblet cells [48]. IL-13 activates Janus kinase 1, which phosphorylates STAT6 after binding to a receptor that contains the IL-4R*α* subunit. Although MUC5AC lacks a consensus STAT6-binding site, STAT6 activation increases the expression of SPDEF, which in turn upregulates numbers of genes involved in mucous metaplasia, and decreases the synthesis of forkhead box A2 (Foxa2), which negatively regulates MUC5AC [49, 50].

**Figure 2:**
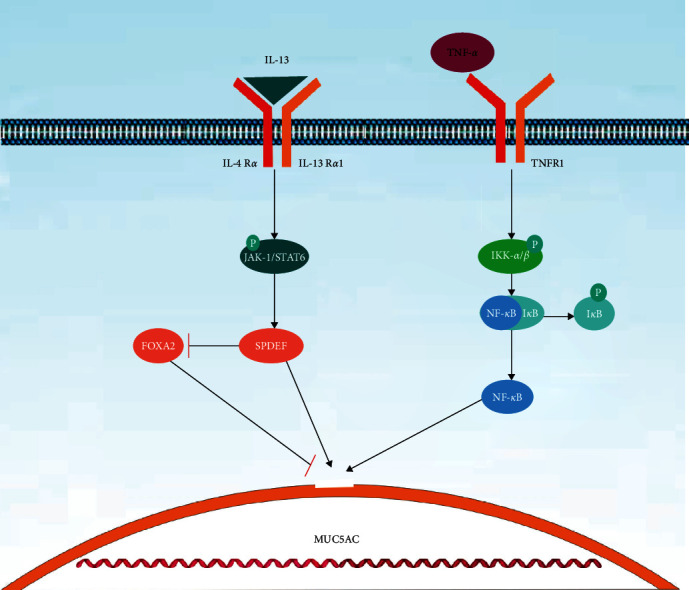
Pathways leading to mucus hypersecretion TNF-*α* interacts with cell surface receptors and stimulates the EGFR gene in airway epithelial cells. I*κ*B kinase (IKK) activation is induced by receptor stimulation. The I*κ*B subunit of intracellular NF-*κ*B is then phosphorylated (P) by IKK at its regulatory site, allowing for transactivation and degradation. This, in turn, releases NF-*κ*B dimers and causes free NF-*κ*B to move into the nucleus, where it activates the expression of target genes MUC5AC. IL-13 activates Janus kinase 1 (Jak1) to phosphorylate (P) STAT6 after binding to IL-4R*α* receptors. STAT6 activation increases the expression of SPDEF, which upregulates mucous metaplasia genes and reduces forkhead box A2 (Foxa2), which negatively regulates MUC5AC.

TNF-*α* binds to cell surface receptors and induces the expression of the EGFR gene in airway epithelial cells, which has been linked to the synthesis of mucin in COPD. I*κ*B kinase (IKK) activation is induced by receptor stimulation. The I*κ*B subunit of intracellular NF-*κ*B is then phosphorylated (P) by IKK at its regulatory site, allowing for transactivation and degradation. This, in turn, releases NF-*κ*B dimers and causes free NF-*κ*B to move into the nucleus, where it activates the expression of target genes such as MUC5AC (Figure 2) [51, 52].

### 2.11. Immune Cells and COPD

The functionality of the immune system is the outcome of the interplay between diverse immune cells and cytokines. T lymphocytes, B lymphocytes, and NK cells are crucial constituents of immune cells. Mature T cells have the ability to differentiate into distinct cell subsets, including CD3 + CD4 + CD8-T and CD3+CD4-CD8+ T-cell subsets. Numerous cytokines are released by activated CD4+ T cells, and these cells control the action of other inflammatory cells by producing perforin, granzyme B, and TNF-*α* to reduce infected cells and help in the degradation and apoptosis of alveolar epithelial cells. Studies show a high correlation between the proportion of CD8+ T cells and the development of COPD. Depending upon the type of cytokines produced, CD4+ T cells produce type 1 T helper (Th1) or Th2 cells. TNF-*α*, IFN-*γ*, and IL-2 are the primary product of Th1 cells, while Th2 cells produce IL-4, IL-10, and IL-16, which stimulates B lymphocyte growth to produce IgG and IgE, which is a key factor in humoral immunity. Studies have shown acute exacerbation of COPD (AECOPD) patients had elevated IL-17 levels, which may encourage neutrophil recruitment and infiltrates into COPD inflammatory areas. Compared to patients with stable COPD, AECOPD exhibits greater levels of IL-2, IFN-*γ*, IL-4, IL-10, IL-17, and IgE, and they have lower Th1/Th2 and Il-17/IgE ratios probably due to increased production of Th2 cells [53, 54].

### 2.12. Innate Lymphoid Cells and COPD

Innate lymphoid cells (ILCs)—ILC1, ILC2, and ILC3, are specialized cells of lymphoid origin that are present in the lung at a steady state and are active contributors to the progression of chronic lung diseases. ILCs are detected in the parenchyma in healthy individuals and also in lymphoid aggregated COPD and smokers. Studies suggest an elevated level of ILC1 in COPD patients correlating the risk of exacerbations, suggesting they could be used as a marker for the disease progression. ILC2s can turn into ILC1s in the context of COPD, indicating a skew into type 1 inflammation. In the setting of smoking-induced COPD, ILC2s play a role in neutrophil recruitment. Their absence protected against emphysema while promoting fibrosis by increasing IL-13 and IL-33 levels. ILC2s have also been linked to the promotion of Th2 adaptive responses during AECOPD [55].

### 2.13. Treatment

The prevalence of cough and expectoration, along with the anticipated role of mucus in the pathogenesis of COPD, leads to develop medications to treat CMH. The main objective of mucus control should be aimed at reducing CMH from the lower respiratory tract and maintain a normal mucus secretion rate and a reasonable viscosity of the mucus to ensure effective clearance. Smoking cessation is a simple but most effective way to reduce excesses mucus production. Bronchodilators, inhaled corticosteroids (ICS), and expectorant therapy have been used to treat mucus hypersecretion to prevent or attenuate airway stenosis and slow down the deterioration of lung function (Table 2).

## 3. Pharmacological Treatment

### 3.1. Hypertonic Saline (HS) Aerosol

Sputum expectoration has traditionally been induced by HS aerosol for diagnostic evaluation as well as for respiratory care [56]. Hypertonic solution inhalation facilitates water movement down the concentration gradient across the water-permeable airway epithelium into the luminal compartment. This additional volume of water is expected to reduce the mucus solid concentration, thus improving the airway secretion transportability. HS can also disrupt ionic bonds and improves MCC [57]. The MCC rate is reduced in CF. Studies have demonstrated that either single or repeated HS inhalation in CF patients can produce long-term effects on MCC and reduce exacerbation [58]. Despite the beneficial effects of HS on CF, no convincing evidence or long-term study of the therapeutic value of HS in COPD has been reported.

### 3.2. Oral Guaifenesin (GGE)

The effects of oral GGE as an expectorant and a cough suppressant have been investigated. It stimulates receptors in the gastrointestinal (GI) mucosa via the gastro-pulmonary reflex. Increased stomach vagal stimulation and consequent vagal activity in the airways may promote MCC [59]. Studies on CB patients have shown that GGE reduces secretion, viscosity, elasticity, mucin synthesis, mucociliary transport, and cough reflex sensitivity [60]. The US Food and Drug Administration has approved GGE as an over-the-counter expectorant [59], but few clinical trial data are available to provide sufficient evidence to support the hypothesis that this oral expectorant can improve the subjective well-being or lung function of COPD patients.

### 3.3. Bronchodilators

The use of bronchodilators has a central role in COPD treatment. Bronchodilator relaxes the airway smooth muscle tone, reduces respiratory muscle activity, and improves ventilation. It lowers airway resistance and inspiratory muscle elastic load during constant work rate exercise. In the peripheral airway, it reduces air trapping and, thus, lung volumes [61]. Currently, three classes of bronchodilators are available: beta_2_-agonist, anticholinergic, and methylxanthines. These can be used alone, or in combination with one another, or with or without ICS.

### 3.4. Beta_2_-Agonists

They act by activating beta_2_ adrenergic receptors, which increase cyclic AMP and provide functional antagonism to bronchoconstriction and are able to relax airway smooth muscles and reduce bronchoconstriction. They also help to improve MCC in COPD and thus reduce the rate of COPD exacerbations [7]. It includes short-acting beta_2_ agonists (SABA) and long-acting beta_2_ agonists (LABA). SABA includes salbutamol and terbutaline and has been used as a rescue medication for COPD with a duration of action of 4–6 hr. So its efficacy is limited for a maintenance dose. Regular and as-needed SABA administration improves both symptoms and FEV1 [62, 63]. On the other hand, LABA is used in stable COPD. It shows the duration of action more than 12 hr. Salmeterol and formoterol are used as two times daily doses, whereas indacaterol and olodaterol, and vilanterol are used as once-daily doses [64]. However, beta_2_-agonists can increase heart rate and produce sinus tachycardia, dose-dependent tremor, and hypokalemia by stimulating Na^+^, K^+^-ATPase-driven pump connected to beta_2_ adrenoreceptors in skeletal muscles. In addition, they induce glycogenolysis and raise blood sugar, and also temporarily lower the partial pressure of arterial oxygen, but the therapeutic importance of these changes is unknown [63, 65].

### 3.5. Anticholinergic

By acting on the muscarinic receptor, anticholinergic is believed to facilitate mucus clearance by increasing luminal sizes and reducing surface and submucosal gland mucin secretion. Muscarinic receptors are mostly present on autonomic effector cells supplied by postganglionic parasympathetic neurons [65, 66]. As many as five distinct muscarinic receptors (M_1_–M_5_) have been identified, of which three (M_1_–M_3_) are present in the lung. Antimuscarinic drugs block acetylcholine's bronchoconstriction actions on the M_3_ receptor in the airway [66]. Ipratropium which is short-acting muscarinic antagonist inhibits M_2_ inhibitory neuronal receptors that contribute to vagally produced bronchoconstriction. Tiotropium, which is a long-acting muscarinic antagonist (LAMA), has a longer duration of binding to M_3_ receptors with a faster duration of dissociation from M_2_ receptors, extending the duration of the bronchodilator effect [67, 68]. Antimuscarinic drug administration frequently causes a foul smell and dry mouth [69]. Older males who use these substances may develop urinary retention. Therefore, these drugs should be taken with caution in individuals who have prostatic hypertrophy or bladder outlet obstruction [62]. Inhaled antimuscarinic medications have been associated with a number of ophthalmic side effects, such as impaired vision, increased intraocular pressure, progressive narrow-angle glaucoma, and cataracts [65]. Possible links between antimuscarinic medications and cardiovascular morbidity and death have given rise to concerns [70, 71].

### 3.6. Methylxanthines

Methylxanthines is a bronchodilator and anti-inflammatory agent that acts by increasing water flux toward the lumen and stimulating the ciliary beat frequency to improve mucus clearance. By increasing cyclic adenosine monophosphate in cells, they can increase the airway lumen diameter, promote cilia motion, and activate transmembrane regulators to induce chloride ion secretion and mucus hydration, resulting in airway cilia transport. Because of its narrow therapeutic window and drug–drug interaction, it is considered a third-line therapy in COPD [72]. Theophylline, a xanthine derivative, has been used to treat COPD for a long time not only because they are affordable and widely available but also because it improves both FEV1 and forced vital capacity in COPD patients as well as increases exercise tolerance [73]. Withdrawal of theophylline produces significant clinical deterioration in patients with severe COPD, despite therapy with alternative bronchodilators, showing its added utility [74]. Nevertheless, recommendations for theophylline in the treatment of COPD differ among national guidelines, most likely due to the fact that it is less effective and tolerated than inhaled long-acting bronchodilators and ICS that show superior anti-inflammatory action [75]. Because of its narrow therapeutic window and the tendency of pharmacological interactions, theophylline is downgraded to the second or third line of therapy, making its usage difficult, particularly in older individuals with comorbidities receiving many classes of medicine [72, 76]. Another xanthine derivative, doxofylline differs structurally from theophylline in that position 7 of the xanthine ring contains a dioxolane group [77]. There is evidence that at least doxofylline is a useful bronchodilator for alleviating airway obstruction and has a safer profile than theophylline, with a favorable risk-to-benefit ratio. These data imply that it may be a feasible option for theophylline in the management of COPD patients [78].

### 3.7. Inhaled Corticosteroids (ICS)

Corticosteroids are a group of potent anti-inflammatory drugs commonly used in the treatment of AECOPD. The use of ICS can suppress IL-13-induced goblet cell metaplasia and reduce MUC5AC and ATP-stimulated mucus secretion, and also restore MUC5B protein levels [79]. ICS therapy is used in half of COPD patients, but its effectiveness in clinical settings is limited. The use of ICS is based on the association between COPD-Asthma overlap syndrome. ICS therapy reduces exacerbation and lowers the course of respiratory symptoms with little or no effect on lung function or mortality. However, ICS is not suggested as monotherapy. A long-term decline in FEV1 and mortality related to COPD have not been reported to be affected by regular use of ICS alone [80, 81]. A randomized clinical trial (RCT) provides more evidence that the use of ICS alters airway microbes and is linked to an increased risk of oral candidiasis, pneumonia, skin bruises, hoarse voice, etc. [81, 82]. Numerous studies have shown a relation between COPD patient's blood eosinophil counts and the effects of ICS. The thresholds of <100 and 300 cells/*µ*l should be viewed as estimated rather than specific cutoff values that can anticipate various probabilities of treatment benefits [83]. In moderate-to-very severe patients, compared to LAMA alone or LABA + LAMA or LABA + ICS, the step-up inhalation therapy with LABA + LAMA + ICS (triple therapy) has been documented to improve lung function with reduced exacerbation rate and patients outcomes [84, 85].

### 3.8. Phosphodiesterase-4 (PDE4) Inhibitors

PDE4 inhibitors are a group of nonsteroidal anti-inflammatory medications that control cough reflexes and prevent mucus secretion from the airway. Roflumilast, a selective PDE4 inhibitor, has been extensively studied for its efficacy and safety in COPD. In addition to use as a baseline therapy, PDE4 reduces neutrophilic and eosinophilic inflammatory mediators in the sputum of COPD patients [86]. A 24 weeks clinical study in Roflumilast-treated patients showed both pre and postbronchodilator FEV1 and QOL were significantly improved as measured by St. George Respiratory Questionnaire [87]. Another multicenter RCT in severe COPD patients who experienced frequent exacerbation showed that the addition of Roflumilast to ICS/LABA or ICS/LABA/LAMA could reduce the severity of COPD exacerbation and the frequency of hospitalization. However, the occurrence of GI adverse events such as diarrhea, weight loss, insomnia, and depressive moods was higher in the Roflumilast treatment group [88, 89].

### 3.9. Macrolide Antibiotics

The cause of airway infection in COPD is multifactorial and bacterial infections are involved in almost half of the cases of COPD, so empirical antibiotics are indicated [90]. Most clinical guidelines suggest a risk stratification approach in selecting antibiotics. Beyond antimicrobial effects, some antibiotics also have mucoactive effects. Macrolide antibiotics are characterized by a macrocytic lactone ring, with better bioavailability and extensive tissue penetration with broad-spectrum antibiotic effects, which makes them effective for treating various respiratory infections. Studies have also shown that macrolide possesses a variety of immune modulatory anti-inflammatory and antiviral effects by reducing mucus secretion and inhibiting bacterial virulence [91]. Macrolides or other antibiotics are not used prophylactically for long-term care of patients with COPD. But for most patients, the benefits of long-term use of antibiotics do not outweigh the risks. However, in selected patients with severe COPD with frequent exacerbation (≥2/year), macrolide prophylactics may be advantageous despite optimal medical treatment. When prescribing a macrolide for long-term prophylaxis, azithromycin 250 mg is usually used daily, or 250–500 mg three times per week. Although the long-term duration is not defined, a 12 month course is commonly used [92, 93]. A systemic review shows the number of patients who experienced ≥1 exacerbation was reduced after the use of prophylactic antibiotics. As a result, their QOL and FEV1 were improved, serious adverse events such as long QT interval and tinnitus were reduced, and the frequency of hospital admission was shortened [90]. The risk associated with the development of antibiotic resistance should be taken into account in deciding long-term prophylaxis with macrolide antibiotics [94].

### 3.10. Ambroxol

Ambroxol is an expectorant with mucociliary activity in addition to its mucokinetic, antioxidant, and anti-inflammatory properties. It can induce the formation of surfactants in alveolar type II cells and provide a local anesthetic effect as well [95]. Some studies have demonstrated its potential to enhance the antibiotic level in the lung tissue and mucus as well as ability to minimize exacerbation in patients in a more severe state when given in conjunction with antibiotics [96, 97]. The use of ambroxol in COPD patients can stimulate sputum clearance, relieve clinical symptoms, and improve lung and immunological functions in AECOPD, thus improving the QOL of the patients and shortening the length of hospital stay [95, 98].

### 3.11. Thiol Derivatives

Thiol derivatives such as *N*-acetylcysteine (NAC), erdosteine, and carbocysteine have typically been viewed as mucolytic medications as they can reduce the viscosity and flexibility of transbronchial secretion by lowering S–S bonds in mucus proteins and acting as a source of replacement for intracellular glutathione level [99]. In addition, they possess antioxidant, anti-inflammatory, and antibacterial properties to improve antibiotic therapy [100]. A meta-analysis study that compared the effectiveness of thiol-based drugs showed mucolytic/antioxidant agents significantly reduced the risk of AECOPD, and the effectiveness was erdosteine > carbocysteine > NAC [101]. Carbocysteine, when available, may be an option for treating frequent COPD exacerbations in patients who are unable to use ICS in combination with LABA or LAMA. However, there is no conclusive evidence that these agents can effectively reduce exacerbations when added to more established inhaled regimens [102].

### 3.12. Novel Therapy

Targeted treatment of pathological airways improves symptoms of cough and dyspnea, decreases the frequency of disease-related exacerbations, and slows down disease progression. But most current treatments mainly aim at relieving symptoms; they face difficulties in clinical efficacy with poor adherence. There is a lack of fully developed therapeutic approaches that can alter metabolism to combat COPD. Novel therapeutic strategies for COPD patients are the subjects of interest and ongoing studies in the future.

### 3.13. Peptides Related to Myristoylated Alanine-Rich C Kinase Substrate (MARCKS)

MARCKS protein has been found to play an important role in airway mucin secretion control. It is a crucial intracellular molecule involved in mucin granule intracellular migration and exocytosis. Studies showed that MARCKS synthesis was inhibited using an antisense oligonucleotide, which reduced both mRNA and protein levels of MARCKS and mucin secretion as well. In addition, MARCKS inhibition was suppressed by a synthetic peptide to its *N*-terminus (MANS peptide) in vitro and in vivo by normal human bronchial epithelial cells and mouse airway epithelial cells [103, 104].

### 3.14. Human Alveolus Retinoic Acid (RA)

Alveolar regeneration is aided by angiogenesis, which is stimulated by either endogenous RA or an exogenous retinoid activating RA signaling in the microvascular endothelium. Several RA receptors (RAR), including RAR-gamma, are studied as inhibitors of alveolar damage. The receptor RAR-alpha, on the other hand, appears to be implicated in mucin expression as well as the establishment and maintenance of hypersecretory phenotype. Some of these functions could be inhibited by RAR-alpha antagonists such as RO-41-5253 [105], and therefore it would be of interest to develop selective RAR-alpha antagonists for the treatment of mucus hypersecretion in respiratory disorders, including COPD [106, 107].

### 3.15. Inhibitors of Phosphodiesterase 5 (PDE5)

PDE5 has been reported to have anti-inflammatory effects. Sildenafil, a powerful PDE5 inhibitor, has been shown to have anti-inflammatory effects on the respiratory tract, goblet cell metaplasia, and the production of MUC5A by cGMP signaling pathway [108].

### 3.16. Calcium-Activated Chloride Ion Channel 1 (CLCA1)

Patients with chronic lung diseases like COPD have goblet cell hyperplasia, which is expressed by the human CLCA1 [109]. Human CLCA 1 inhibition reduces mucin expression. Nilumic acid, a relatively specific CLCA inhibitor, has been shown to inhibit human CLCA 1 and reduce the expression of mucin in both animal and human airway mucosa [110].

### 3.17. Immune Cell Therapy (ICT)

The potential of ICT as a novel treatment for COPD is under investigation. The field of ICT refers to regulating the immune system and reducing lung inflammation through the utilization of immune cells, including macrophages, regulatory T cells, and NK cells. The safety and efficacy of these medications have been demonstrated in animal testing. A study shows ACT can greatly enhance the level of immune cells in the body while maintaining it for a long time. However, more studies are necessary to determine their therapeutic potential in more human clinical trials in COPD [54].

### 3.18. EGFR Tyrosine Kinase Inhibitor

Through the EGFR pathway, NE and ROS can cause mucus hypersecretion leading to exacerbation of COPD associated with a decline in FEV1 [43]. The role of selective EGFR tyrosine kinase inhibitors has been studied in the treatment of mucus hypersecretion in vivo (BIBX 1522) [111, 112]. Human trials are necessary to define the role of EGFR inhibitors in the treatment of mucus hypersecretion.

### 3.19. Surfactants

The lubricating properties of surfactant between the mucus gel and periciliary sol layer facilitate the transfer of energy from cilia beating to mucus. Although the use of surfactants is still experimental, aerosolized surfactants in stable CB patients show an increase in sputum transport by cilia and an improvement in pulmonary function [113, 114].

### 3.20. Interventional Bronchoscopy Therapy

Despite extensive treatment, symptoms of CB progress over time, and it is challenging to treat them. Numerous experimental bronchoscopy therapies are recently studied, but larger studies are required to validate the benefits of this interventional therapy which are still in the early stages of clinical testing. A more significant idea of this therapy evaluation has revealed encouraging results on the frequency of exacerbation [115, 116]. Some of the bronchoscopy therapies are noted in Table 3.

## 4. Nonpharmacological Treatment

### 4.1. Exercise in COPD

Patients with COPD often have a disproportionate metabolic cost of performance compared to healthy people of the same age, including early onset lactic acidosis and decreased optimum effort and oxygen consumption. Patients' QOL is often adversely affected since they are confined to their homes and are unable to engage in their usual activities of everyday life. However, early research from the 1990s showed that patients with COPD who engaged in high-intensity training showed improvements in their skeletal muscle oxidative capacity and exercise-induced lactic acidosis. Low-intensity training is safe for those with COPD and is recommended for individuals with more advanced diseases [117]. According to the American Lung Association, COPD patients should engage in (1) pulmonary rehabilitation (programs that teach about lungs and disease and how to exercise and be more active while experiencing less shortness of breath), (2) stretching, (3) aerobic exercises like walking, riding, and swimming, and (4) resistance training [118].

### 4.2. Smoking Cessation

Cutting down the number of cigarette smoking is one of the prime interventions that should be initiated by patients themselves as well as policymakers should implement new strategies for it [119]. It is the most cost-effective method to prevent lung function deterioration in COPD patients [120].

### 4.3. Chest Physiotherapy

Chest physiotherapy is an essential component of COPD management. For individuals with severe COPD, chest physiotherapy may provide further clinical benefits [121]. Over the years, numerous methods have been proposed for treating COPD, including postural drainage, forced expiratory technique, assisted coughing, oscillatory positive-expiratory pressure devices, autogenic drainage, high-frequency chest wall oscillation, intrapulmonary percussive ventilation, intermittent positive pressure breathing, and temporary positive expiratory pressure. All these methods aim to clear secretions of the bronchial tree [122]. The question of whether or not these methods are effective during an acute exacerbation or stable disease remains unclear.

## 5. Conclusion

COPD is one of the major causes of morbidity and mortality in the world, imposing an expanded financial and social burden. The prevalence of COPD is directly related to the rate of smoking in the country and among different groups, though environmental air pollution is additionally likewise a sizeable hazard issue. Due to solid continuing exposure to risk factors and the aging of the population, the burden and prevalence of COPD will increase in the coming decades. Overproduction and hypersecretion of goblet cells and a decreased elimination of mucus are reasons behind hypersecretion leading to exacerbation and decreased QOL. Given the consensus that mucus hypersecretion contributes significantly to the pathophysiology of chronic airway diseases, inhibition of CMH should be a therapeutic focus in future research. The conventional guidelines, including both pharmacological and nonpharmacological treatment, are not fully effective. Therefore, it is ideal to optimize the strongest possible results aiming at developing effective molecules as well as interventional procedures that can hinder pathophysiological pathways and explore strategies to prevent and treat disease exacerbation.

## Figures and Tables

**Table 1:** Types of mucins and their chromosome locations and names.

Mucin symbols	Chromosome location	Previous symbols	Mucin name
MUC1	1q22	PMU, MCKD1	Cell surface associated, mucin 1
MUC2	11p15.5		Oligomeric mucus/gel-forming, mucin 2
MUC3A	7q22.1	MCU3	Cell surface associated, mucin 3A
MUC3B	7q22		Cell surface associated, mucin 3B
MUC4	3q29		Cell surface associated, mucin 4
MUC5AC	11p15.5		Oligomeric mucus/gel-forming, mucin 5AC
MUC5B	11p15.5	MCU5	Oligomeric mucus/gel-forming, mucin 5B
MUC6	11p15.5		Oligomeric mucus/gel-forming, mucin 6
MUC7	4q13.3		Secreted, mucin 7
MUC8	12q24.33		Mucin 8
OVGP1	1p13.2	MCU9	Oviductal glycoprotein 1
MUC12	7q22.1	MCU11	Cell surface associated, mucin 12
MUC13	3q21.2	DRCC1	Cell surface associated, mucin 13
EMCN	4q24		Endomucin
MUC15	11p14.2		Cell surface associated, mucin 15
MUC16	19p13.2		Cell surface associated, mucin 16
MUC17	7q22.1		Cell surface associated, mucin 17
MUC19	12q12		Oligomeric, mucin 19
MUC20	3q29		Cell surface associated, mucin 20
MUC21	6p21.33	C6orf205	Cell surface associated, mucin 21
MUC22	6p21.33		Mucin 22

**Table 2:** Pharmacotherapy of COPD.

Therapy	Mechanism of action	Drug name	Adverse effects	Note
Inhaled short-acting *β*2-agonist (SABA)	Stimulates *β*2 adrenergic receptors, increasing cyclic AMP with functional antagonism to bronchoconstriction	Albuterol (salbutamol), levalbuterol	Sinus tachycardia, dose-dependent tremors, and hypokalemia, temporarily lower partial pressure of arterial oxygen	In most cases, used for mild diseases with low symptoms or more advanced diseases to relieve acute symptoms in addition to maintenance inhalers
Inhaled long-acting *β*2-agonist (LABA)		Salmeterol, formoterol, indacaterol, olodaterol, vilanterol	Headache, sinus tachycardia, dose-dependent tremor, and hypokalemia, temporarily lower partial pressure of arterial oxygen	Selected as maintenance therapy when short-acting bronchodilators do not give adequate relief of symptoms
Inhaled short-acting muscarinic antagonist (SAMA)	Blocks bronchoconstriction effects of acetylcholine on M3 muscarinic receptors expressed in airway smooth muscles	Ipratropium bromide, oxitropium bromide	Foul smell, dry mouth, urinary retention, impaired vision, progressive narrow-angle glaucoma, and cataracts	In most cases, used for mild diseases with low symptoms or more advanced diseases to relieve acute symptoms in addition to maintenance inhalers
Inhaled long-acting muscarinic antagonist (LAMA)		Tiotropium bromide, aclidinium bromide, glycopyrronium bromide, umeclidinium, glycopyrroate, revefenacin	Foul smell, dry mouth, urinary retention, impaired vision, progressive narrow-angle glaucoma, and cataracts	Selected as maintenance therapy when short-acting bronchodilators do not give adequate relief of symptoms, also in those who are at risk of exacerbations
Methylxanthine	Inhibition of cyclic nucleotide phosphodiesterases with competitive antagonism of adenosine receptors with bronchodilator and anti-inflammatory effects	Theophylline, doxofylline	Headache, restlessness, insomnia, nausea, vomiting, cardiac flutter, tachycardia, seizure, and status epilepticus with higher doses	Not suggested unless other long-acting bronchodilators are either intolerable or costly
Inhaled corticosteroids	Anti-inflammatory and suppression of IL-13-induced goblet cell metaplasia and reduce MUC5AC and ATP-stimulated mucus secretion	Fluticasone, budesonide, beclomethasone	Oral candidiasis, pneumonia, skin bruises, hoarse voice	Typically combined with a long-acting bronchodilator rather than administered alone in patients at risk for exacerbation
Phosphodiesterase-4 inhibitors	Selective inhibitors of PDE-4 inhibiting the breakdown of intracellular cyclic AMP with anti-inflammatory effects	Roflumilast	Diarrhea, weight loss, insomnia, and depressive moods	In patients with moderate to severe COPD, it is recommended as a maintenance therapy for the prevention of exacerbations
Macrolide antibiotics	Reduce airway mucus secretion with anti-inflammatory effects	Azithromycin	Nausea, diarrhea, abdominal pain	Recommended as a maintenance therapy for the prevention of exacerbations
Ambroxol	Expectorant with mucokinetic, antioxidant, and anti-inflammatory properties and can induce surfactant formation with inhibition of neuronal sodium channels	Ambroxol	Occasional GI side effects but are mild in nature	As an expectorant with antioxidative and anti-inflammatory properties, it enhances antibiotic levels in the lung tissue and mucus and minimizes exacerbation when given with antibiotics
Thiol derivatives	Mucolytic agents act by lowering S–S bonds with antioxidant, anti-inflammatory properties	*N*-acetylcysteine (NAC), erdosteine, carbocysteine	Nausea, vomiting, foul smell, dry mouth	As mucolytic agents with antioxidants, anti-inflammatory, and antibacterial properties, it reduces mucus viscosity by lowering S–S bonds in mucus proteins and acts as a source of replacement for intracellular glutathione level

**Table 3:** Interventional bronchoscopy procedure.

Interventional bronchoscopy procedure	Aim
Bronchial rheoplasty	Reduces symptoms and goblet cell hyperplasia by delivering nonthermal pulsed electric fields to mucus-producing airway cells
Resector balloon desobstruction	Through bronchial lumen, a resector latex balloon is inflated and deflated causing disruption of hyperplastic goblet cells
Targeted lung denervation (TLD)	Bronchoscopy radiofrequency ablation is used to disrupt pulmonary parasympathetic nervous system, which is responsible for airway inflammation and mucus hypersecretion
Spray cryotherapy	Uses nitrogen spray, which can induce airway tissue repair without scaring by destroying hyperplastic goblet cells and excess submucosal glands

## References

[1] Sandelowsky H., Weinreich U. M., Aarli B. B. (2021). COPD – do the right thing. *BMC Family Practice*.

[2] World Health Organization (March 2022). Chronic obstructive pulmoanry disease (COPD). https://www.who.int/news-room/fact-sheets/detail/chronic-obstructive-pulmonary-disease-(copd).

[3] (November 2023). Global initiative for chronic obstructive lung disease: 2023 Report. https://goldcopd.org/2023-gold-report-2/.

[4] Institute for Health Metrics and Evaluation (August 2017). Chronic obstructive pulmonary disease caused 3.2 million deaths worldwide in 2015. https://www.healthdata.org/news-release/chronic-obstructive-pulmonary-disease-caused-32-million-deaths-worldwide-2015.

[5] Yang T., Cai B., Cao B. (2022). Severity distribution and treatment of chronic obstructive pulmonary disease in China: baseline results of an observational study. *Respiratory Research*.

[6] Kim V., Oros M., Durra H. (2015). Chronic bronchitis and current smoking are associated with more goblet cells in moderate to severe COPD and smokers without airflow obstruction. *PLOS ONE*.

[7] Rodrigues S. d. O., Cunha C. M. C. d., Soares G. M. V., Silva P. L., Silva A. R., Gonçalves-de-Albuquerque C. F. (2021). Mechanisms, pathophysiology and currently proposed treatments of chronic obstructive pulmonary disease. *Pharmaceuticals*.

[8] Whitsett J. A., Alenghat T. (2015). Respiratory epithelial cells orchestrate pulmonary innate immunity. *Nature Immunology*.

[9] Kesimer M., Kirkham S., Pickles R. J. (2009). Tracheobronchial air-liquid interface cell culture: a model for innate mucosal defense of the upper airways?. *American Journal of Physiology-Lung Cellular and Molecular Physiology*.

[10] Williams O. W., Sharafkhaneh A., Kim V., Dickey B. F., Evans C. M. (2006). Airway mucus: from production to secretion. *American Journal of Respiratory Cell and Molecular Biology*.

[11] Bansil R., Turner B. S. (2006). Mucin structure, aggregation, physiological functions and biomedical applications. *Current Opinion in Colloid & Interface Science*.

[12] Lamblin G., Aubert J. P., Perini J. M. (1992). Human respiratory mucins. *European Respiratory Journal*.

[13] Rose M. C. (1992). Mucins: structure, function, and role in pulmonary diseases. *American Journal of Physiology-Lung Cellular and Molecular Physiology*.

[14] Silberberg A. (1989). Mucus glycoprotein, its biophysical and gel-forming properties. *Symposia of the Society for Experimental Biology*.

[15] Fahy J. V., Dickey B. F. (2010). Airway mucus function and dysfunction. *New England Journal of Medicine*.

[16] Bustamante-Marin X. M., Ostrowski L. E. (2017). Cilia and mucociliary clearance. *Cold Spring Harbor Perspectives in Biology*.

[17] Ma J., Rubin B. K., Voynow J. A. (2018). Mucins, mucus, and goblet cells. *Chest*.

[18] Mitran S. (2013). Continuum-kinetic-microscopic model of lung clearance due to core-annular fluid entrainment. *Journal of Computational Physics*.

[19] Fröhlich E. (2022). Non-cellular layers of the respiratory tract: protection against pathogens and target for drug delivery. *Pharmaceutics*.

[20] Wagner C. E., Wheeler K. M., Ribbeck K. (2018). Mucins and their role in shaping the functions of mucus barriers. *Annual Review of Cell and Developmental Biology*.

[21] HUGO Gene Nomenclature Committee (HGNC) (2018). Gene group: Mucins (MUC). https://www.genenames.org/data/genegroup/#!/group/648.

[22] Hattrup C. L., Gendler S. J. (2008). Structure and function of the cell surface (tethered) mucins. *Annual Review of Physiology*.

[23] Thornton D. J., Rousseau K., McGuckin M. A. (2008). Structure and function of the polymeric mucins in airways mucus. *Annual Review of Physiology*.

[24] Boucher R. C. (2019). Muco-obstructive lung diseases. *New England Journal of Medicine*.

[25] Okuda K., Chen G., Subramani D. B. (2019). Localization of secretory mucins MUC5AC and MUC5B in normal/healthy human airways. *American Journal of Respiratory and Critical Care Medicine*.

[26] Song D., Iverson E., Kaler L., Boboltz A., Scull M. A., Duncan G. A. (2022). MUC5B mobilizes and MUC5AC spatially aligns mucociliary transport on human airway epithelium. *Science Advances*.

[27] Hogg J. C., Chu F. S. F., Tan W. C. (2007). Survival after lung volume reduction in chronic obstructive pulmonary disease: insights from small airway pathology. *American Journal of Respiratory and Critical Care Medicine*.

[28] Milnerowicz H., Ściskalska M., Dul M. (2015). Molecular mechanisms of the impact of smoke-oxidants. *Experimental and Toxicologic Pathology*.

[29] Rahman I., Morrison D., Donaldson K., MacNee W. (1996). Systemic oxidative stress in asthma, COPD, and smokers. *American Journal of Respiratory and Critical Care Medicine*.

[30] Rahman I., MacNee W. (2000). Regulation of redox glutathione levels and gene transcription in lung inflammation: therapeutic approaches. *Free Radical Biology and Medicine*.

[31] Deshmukh H. S., Shaver C., Case L. M. (2008). Acrolein-activated matrix metalloproteinase 9 contributes to persistent mucin production. *American Journal of Respiratory Cell and Molecular Biology*.

[32] Li J.-D., Dohrman A. F., Gallup M. (1997). Transcriptional activation of mucin by *Pseudomonas aeruginosa* lipopolysaccharide in the pathogenesis of cystic fibrosis lung disease. *Proceedings of the National Academy of Sciences*.

[33] McNamara N., Basbaum C. (2001). Signaling networks controlling mucin production in response to Gram-positive and Gram-negative bacteria. *Glycoconjugate Journal*.

[34] Jono H., Xu H., Kai H. (2003). Transforming growth factor-*β*-smad signaling pathway negatively regulates nontypeable *Haemophilus influenzae*-induced MUC5AC mucin transcription via mitogen-activated protein kinase (MAPK) Phosphatase-1-dependent Inhibition of p38 MAPK. *Journal of Biological Chemistry*.

[35] Zhao C.-Z., Fang X.-C., Wang D., Tang F.-D., Wang X.-D. (2010). Involvement of type II pneumocytes in the pathogenesis of chronic obstructive pulmonary disease. *Respiratory Medicine*.

[36] Ruaro B., Salton F., Braga L. (2021). The history and mystery of alveolar epithelial type II cells: focus on their physiologic and pathologic role in lung. *International Journal of Molecular Sciences*.

[37] Verberne L. D. M., Leemrijse C. J., Swinkels I. C. S., van Dijk C. E., de Bakker D. H., Nielen M. M. J. (2017). Overweight in patients with chronic obstructive pulmonary disease needs more attention: a cross-sectional study in general practice. *npj Primary Care Respiratory Medicine*.

[38] Spelta F., Fratta Pasini A. M., Cazzoletti L., Ferrari M. (2018). Body weight and mortality in COPD: focus on the obesity paradox. *Eating and Weight Disorders - Studies on Anorexia, Bulimia and Obesity*.

[39] Bruzzaniti S., Bocchino M., Santopaolo M. (2019). An immunometabolic pathomechanism for chronic obstructive pulmonary disease. *Proceedings of the National Academy of Sciences*.

[40] Stănescu D., Sanna A., Veriter C. (1996). Airways obstruction, chronic expectoration, and rapid decline of FEV1 in smokers are associated with increased levels of sputum neutrophils. *Thorax*.

[41] Kao S. S.-T., Ramezanpour M., Bassiouni A., Wormald P.-J., Psaltis A. J., Vreugde S. (2019). The effect of neutrophil serine proteases on human nasal epithelial cell barrier function. *International Forum of Allergy & Rhinology*.

[42] Fischer B., Voynow J. (2000). Neutrophil elastase induces MUC5AC messenger RNA expression by an oxidant-dependent mechanism. *Chest*.

[43] Kohri K., Ueki I. F., Nadel J. A. (2002). Neutrophil elastase induces mucin production by ligand-dependent epidermal growth factor receptor activation. *American Journal of Physiology-Lung Cellular and Molecular Physiology*.

[44] Chung K. F. (2005). Inflammatory mediators in chronic obstructive pulmonary disease. *Current Drug Target -Inflammation & Allergy*.

[45] Izuhara K., Ohta S., Shiraishi H. (2009). The mechanism of mucus production in bronchial asthma. *Current Medicinal Chemistry*.

[46] Wu M., Lai T., Jing D. (2021). Epithelium-derived IL17A promotes cigarette smoke-induced inflammation and mucus hyperproduction. *American Journal of Respiratory Cell and Molecular Biology*.

[47] Cheng X.-H., Black M., Ustiyan V. (2014). SPDEF inhibits prostate carcinogenesis by disrupting a positive feedback loop in regulation of the Foxm1 oncogene. *Plos Genetics*.

[48] Park K.-S., Korfhagen T. R., Bruno M. D. (2007). SPDEF regulates goblet cell hyperplasia in the airway epithelium. *Journal of Clinical Investigation*.

[49] Chen G., Korfhagen T. R., Xu Y. (2009). SPDEF is required for mouse pulmonary goblet cell differentiation and regulates a network of genes associated with mucus production. *Journal of Clinical Investigation*.

[50] Wan H., Kaestner K. H., Ang S.-L. (2004). Foxa2 regulates alveolarization and goblet cell hyperplasia. *Development*.

[51] Lee S. U., Lee S., Ro H. (2018). Piscroside C inhibits TNF-*α*/NF-*κ*B pathway by the suppression of PKC*δ* activity for TNF-RSC formation in human airway epithelial cells. *Phytomedicine*.

[52] Li J., Ye Z. (2020). The potential role and regulatory mechanisms of MUC5AC in chronic obstructive pulmonary disease. *Molecules*.

[53] Wei B., Sheng Li C. (2018). Changes in Th1/Th2-producing cytokines during acute exacerbation chronic obstructive pulmonary disease. *Journal of International Medical Research*.

[54] Li W., Li G., Zhou W., Wang H., Zheng Y. (2022). Effect of autoimmune cell therapy on immune cell content in patients with COPD: a randomized controlled trial. *Computational and Mathematical Methods in Medicine*.

[55] Hsu A. T., Gottschalk T. A., Tsantikos E., Hibbs M. L. (2021). The role of innate lymphoid cells in chronic respiratory diseases. *Frontiers in Immunology*.

[56] Huijghebaert S., Hoste L., Vanham G. (2021). Essentials in saline pharmacology for nasal or respiratory hygiene in times of COVID-19. *European Journal of Clinical Pharmacology*.

[57] Terlizzi V., Masi E., Francalanci M., Taccetti G., Innocenti D. (2021). Hypertonic saline in people with cystic fibrosis: review of comparative studies and clinical practice. *Italian Journal of Pediatrics*.

[58] Wark P., McDonald V. M. (2018). Nebulised hypertonic saline for cystic fibrosis. *Cochrane Database of Systematic Reviews*.

[59] Ohar J. A., Donohue J. F., Spangenthal S. (2019). The role of guaifenesin in the management of chronic mucus hypersecretion associated with stable chronic bronchitis: a comprehensive review. *Chronic Obstructive Pulmonary Diseases*.

[60] Albrecht H. H., Dicpinigaitis P. V., Guenin E. P. (2017). Role of guaifenesin in the management of chronic bronchitis and upper respiratory tract infections. *Multidisciplinary Respiratory Medicine*.

[61] Matera M. G., Page C. P., Calzetta L., Rogliani P., Cazzola M. (2020). Pharmacology and therapeutics of bronchodilators revisited. *Pharmacological Reviews*.

[62] Cazzola M., Matera M. G. (2014). Bronchodilators: current and future. *Clinics in Chest Medicine*.

[63] Cazzola M., Page C. P., Rogliani P., Gabriella Matera M. (2013). *β*2-agonist therapy in lung disease. *American Journal of Respiratory and Critical Care Medicine*.

[64] Fuso L., Mores N., Valente S., Malerba M., Montuschi P. (2013). Long-acting beta-agonists and their association with inhaled corticosteroids in COPD. *Current Medicinal Chemistry*.

[65] Cazzola M., Page C. P., Calzetta L., Gabriella Matera M., Sibley D. R. (2012). Pharmacology and therapeutics of bronchodilators. *Pharmacological Reviews*.

[66] Cazzola M., Page C., Matera M. G. (2013). Long-acting muscarinic receptor antagonists for the treatment of respiratory disease. *Pulmonary Pharmacology & Therapeutics*.

[67] Papi A., Fabbri L. M., Kerstjens H. A. M., Rogliani P., Watz H., Singh D. (2021). Inhaled long-acting muscarinic antagonists in asthma—a narrative review. *European Journal of Internal Medicine*.

[68] Alagha K., Palot A., Sofalvi T. (2014). Long-acting muscarinic receptor antagonists for the treatment of chronic airway diseases. *Therapeutic Advances in Chronic Disease*.

[69] Anzueto A., Miravitlles M. (2020). Tiotropium in chronic obstructive pulmonary disease—a review of clinical development. *Respiratory Research*.

[70] Michele T. M., Pinheiro S., Iyasu S. (2010). The safety of tiotropium—The FDA’s conclusions. *New England Journal of Medicine*.

[71] Anthonisen N. R., Connett J. E., Enright P. L., Manfreda J. (2002). Hospitalizations and mortality in the lung health study. *American Journal of Respiratory and Critical Care Medicine*.

[72] Spina D., Page C. P., Page C., Barnes P. (2017). Xanthines and phosphodiesterase inhibitors. *Pharmacology and Therapeutics of Asthma and COPD*.

[73] Barnes P. J. (2013). Theophylline. *American Journal of Respiratory and Critical Care Medicine*.

[74] Kirsten D. K., Wegner R. E., Jorres R. A., Magnussen H. (1993). Effects of theophylline withdrawal in severe chronic obstructive pulmonary disease. *Chest*.

[75] Cazzola M., Calzetta L., Barnes P. J. (2018). Efficacy and safety profile of xanthines in COPD: a network meta-analysis. *European Respiratory Review*.

[76] Horita N., Miyazawa N., Kojima R., Inoue M., Ishigatsubo Y., Kaneko T. (2016). Chronic use of theophylline and mortality in chronic obstructive pulmonary disease: a meta-analysis. *Archivos de Bronconeumología (English Edition)*.

[77] Franzone J. S., Cirillo R., Barone D. (1988). Doxofylline and theophylline are xanthines with partly different mechanisms of action in animals. *Drugs Under Experimental and Clinical Research*.

[78] Cazzola M., Calzetta L., Rogliani P., Page C., Matera M. G. (2018). Impact of doxofylline in COPD: a pairwise meta-analysis. *Pulmonary Pharmacology & Therapeutics*.

[79] Lachowicz-Scroggins M. E., Finkbeiner W. E., Gordon E. D. (2017). Corticosteroid and long-acting *β*-agonist therapy reduces epithelial goblet cell metaplasia. *Clinical & Experimental Allergy*.

[80] Nici L., Mammen M. J., Charbek E. (2020). Pharmacologic management of chronic obstructive pulmonary disease. An official American Thoracic Society Clinical Practice Guideline. *American Journal of Respiratory and Critical Care Medicine*.

[81] Yang I. A., Clarke M. S., Sim E. H. A., Fong K. M. (2012). Inhaled corticosteroids for stable chronic obstructive pulmonary disease. *Cochrane Database of Systematic Reviews*.

[82] Filho F. S. L., Takiguchi H., Akata K. (2021). Effects of inhaled corticosteroid/long-acting *β*_2_-agonist combination on the airway microbiome of patients with chronic obstructive pulmonary disease: a randomized controlled clinical trial (DISARM). *American Journal of Respiratory and Critical Care Medicine*.

[83] Singh D., Agusti A., Martinez F. J. (2022). Blood eosinophils and chronic obstructive pulmonary disease: a global initiative for chronic obstructive lung disease science committee 2022 review. *American Journal of Respiratory and Critical Care Medicine*.

[84] Lipson D. A., Barnhart F., Brealey N. (2018). Once-daily single-inhaler triple versus dual therapy in patients with COPD. *New England Journal of Medicine*.

[85] Koarai A., Yamada M., Ichikawa T., Fujino N., Kawayama T., Sugiura H. (2021). Triple versus LAMA/LABA combination therapy for patients with COPD: a systematic review and meta-analysis. *Respiratory Research*.

[86] Yuan L., Dai X., Yang M., Cai Q., Shao N. (2016). Potential treatment benefits and safety of roflumilast in COPD: a systematic review and meta-analysis. *International Journal of Chronic Obstructive Pulmonary Disease*.

[87] Rabe K. F., Bateman E. D., O’Donnell D., Witte S., Bredenbröker D., Bethke T. D. (2005). Roflumilast—an oral anti-inflammatory treatment for chronic obstructive pulmonary disease: a randomised controlled trial. *The Lancet*.

[88] Janjua S., Fortescue R., Poole P. (2020). Phosphodiesterase-4 inhibitors for chronic obstructive pulmonary disease. *The Cochrane database of systematic reviews*.

[89] Martinez F. J., Rabe K. F., Sethi S. (2016). Effect of roflumilast and inhaled corticosteroid/long-acting *β*_2_-agonist on chronic obstructive pulmonary disease exacerbations (RE(2)SPOND). A randomized clinical trial. *American Journal of Respiratory and Critical Care Medicine*.

[90] Herath S. C., Normansell R., Maisey S., Poole P. (2018). Prophylactic antibiotic therapy for chronic obstructive pulmonary disease (COPD). *Cochrane Database of Systematic Reviews*.

[91] Yamaya M., Azuma A., Takizawa H., Kadota J.-I., Tamaoki J., Kudoh S. (2012). Macrolide effects on the prevention of COPD exacerbations. *European Respiratory Journal*.

[92] Uzun S., Djamin R. S., Kluytmans J. A. J. W. (2014). Azithromycin maintenance treatment in patients with frequent exacerbations of chronic obstructive pulmonary disease (COLUMBUS): a randomised, double-blind, placebo-controlled trial. *The Lancet Respiratory Medicine*.

[93] Pomares X., Montón C., Espasa M., Casabon J., Monsó E., Gallego M. (2011). Long-term azithromycin therapy in patients with severe COPD and repeated exacerbations. *International Journal of Chronic Obstructive Pulmonary Disease*.

[94] Cui Y., Luo L., Li C., Chen P., Chen Y. (2018). Long-term macrolide treatment for the prevention of acute exacerbations in COPD: a systematic review and meta-analysis. *International Journal of Chronic Obstructive Pulmonary Disease*.

[95] Malerba M., Ragnoli B. (2008). Ambroxol in the 21st century: pharmacological and clinical update. *Expert Opinion on Drug Metabolism & Toxicology*.

[96] Deretic V., Timmins G. S. (2019). Enhancement of lung levels of antibiotics by ambroxol and bromhexine. *Expert Opinion on Drug Metabolism & Toxicology*.

[97] Peralta J., Poderoso J. J., Corazza C., Fernández M., Guerreiro R. B., Wiemeyer J. C. (1987). Ambroxol plus amoxicillin in the treatment of exacerbations of chronic bronchitis. *Arzneimittel-Forschung*.

[98] Li Z. (2021). The effect of adjuvant therapy with ambroxol hydrochloride in elderly chronic obstructive pulmonary disease patients. *American Journal of Translational Research*.

[99] Aldini G., Altomare A., Baron G. (2018). N-Acetylcysteine as an antioxidant and disulphide breaking agent: the reasons why. *Free Radical Research*.

[100] Poole P., Black P. N., Cates C. J. (2012). Cochrane Database of Systematic Reviews. *Cochrane Database Syst Rev*.

[101] Rogliani P., Matera M. G., Page C., Puxeddu E., Cazzola M., Calzetta L. (2019). Efficacy and safety profile of mucolytic/antioxidant agents in chronic obstructive pulmonary disease: a comparative analysis across erdosteine, carbocysteine, and N-acetylcysteine. *Respiratory Research*.

[102] Poole P., Sathananthan K., Fortescue R. (2019). Mucolytic agents versus placebo for chronic bronchitis or chronic obstructive pulmonary disease. *Cochrane Database of Systematic Reviews*.

[103] Li Y., Martin L. D., Spizz G., Adler K. B. (2001). MARCKS protein is a key molecule regulating mucin secretion by human airway epithelial cells in vitro. *Journal of Biological Chemistry*.

[104] Singer M., Martin L. D., Vargaftig B. B. (2004). A MARCKS-related peptide blocks mucus hypersecretion in a mouse model of asthma. *Nature Medicine*.

[105] Apfel C., Bauer F., Crettaz M. (1992). A retinoic acid receptor alpha antagonist selectively counteracts retinoic acid effects. *Proceedings of the National Academy of Sciences*.

[106] Ng-Blichfeldt J.-P., Alçada J., Angeles Montero M. (2017). Deficient retinoid-driven angiogenesis may contribute to failure of adult human lung regeneration in emphysema. *Thorax*.

[107] Ng-Blichfeldt J.-P., Schrik A., Kortekaas R. K. (2018). Retinoic acid signaling balances adult distal lung epithelial progenitor cell growth and differentiation. *EBioMedicine*.

[108] Wang T., Liu Y., Chen L. (2009). Effect of sildenafil on acrolein-induced airway inflammation and mucus production in rats. *European Respiratory Journal*.

[109] Hauber H. P., Tsicopoulos A., Wallaert B. (2004). Expression of HCLCA1 in cystic fibrosis lungs is associated with mucus overproduction. *European Respiratory Journal*.

[110] Liu C.-L., Shi G.-P. (2019). Calcium-activated chloride channel regulator 1 (CLCA1): more than a regulator of chloride transport and mucus production. *World Allergy Organization Journal*.

[111] Takeyama K., Dabbagh K., Jeong Shim J. J., Dao-Pick T., Ueki I. F., Nadel J. A. (2000). Oxidative stress causes mucin synthesis via transactivation of epidermal growth factor receptor: role of neutrophils. *The Journal of Immunology*.

[112] Kim S., Nadel J. A. (2004). Role of neutrophils in mucus hypersecretion in COPD and implications for therapy. *Treatments in Respiratory Medicine*.

[113] Günaydın F. E., Günlüoğlu G., Kalkan N., Aktepe E. N., Demirkol B., Altın S. (2019). The relationship between serum levels of surfactant protein D in COPD exacerbation severity and mortality. *Turkish Journal of Medical Sciences*.

[114] Anzueto A., Jubran A., Ohar J. A. (1997). Effects of aerosolized surfactant in patients with stable chronic bronchitis: a prospective randomized controlled trial. *JAMA*.

[115] Hartman J. E., Garner J. L., Shah P. L., Slebos D.-J. (2021). New bronchoscopic treatment modalities for patients with chronic bronchitis. *European Respiratory Review*.

[116] Duan H., Li X., Long X., Liu X., Wang C., Xie S. (2021). A pilot study of spray cryotherapy effects on airway secretions. *Cryobiology*.

[117] Fiorentino G., Esquinas A. M., Annunziata A., Xiao J. (2020). Exercise and chronic obstructive pulmonary disease (COPD). *Physical Exercise for Human Health*.

[118] American Lung Association (2023). Physical Activity and COPD. https://www.lung.org/lung-health-diseases/lung-disease-lookup/copd/living-with-copd/physical-activity.

[119] Chan S. S. C., Cheung Y. T. D., Wong D. C. N. (2019). Promoting smoking cessation in China: a foot-in-the-door approach to tobacco control advocacy. *Global Health Promotion*.

[120] Bai J.-W., Chen X. X., Liu S., Yu L., Xu J. F. (2017). Smoking cessation affects the natural history of COPD. *International Journal of Chronic Obstructive Pulmonary Disease*.

[121] Dimitrova A., Izov N., Maznev I., Vasileva D., Nikolova M. (2017). Physiotherapy in patients with chronic obstructive pulmonary disease. *Open Access Macedonian Journal of Medical Sciences*.

[122] Nicolini A., Grecchi B., Ferrari-Bravo M., Barlascini C. (2018). Safety and effectiveness of the high-frequency chest wall oscillation vs intrapulmonary percussive ventilation in patients with severe COPD. *International Journal of Chronic Obstructive Pulmonary Disease*.

